# Bioactive Nitrogenous Secondary Metabolites from the Marine Sponge Genus *Haliclona*

**DOI:** 10.3390/md17120682

**Published:** 2019-12-03

**Authors:** Jiaying Zhu, Yang Liu, Zijun Liu, Hao Wang, Huawei Zhang

**Affiliations:** School of Pharmaceutical Sciences, Zhejiang University of Technology, Hangzhou 310014, China; qh.zhujiaying@57qingong.com (J.Z.); liu1395719032@163.com (Y.L.); liuzijun19991108@sina.com (Z.L.); hao_wang_3@163.com (H.W.)

**Keywords:** marine sponge, *Haliclona*, symbiotic microbe, secondary metabolite, alkaloid, bioactivity

## Abstract

Marine sponge genus *Haliclona*, one of the most prolific sources of natural products, contains over 600 species but only a small part of them had been classified and chemically investigated. On the basis of extensive literature search, this review firstly summarizes 112 nitrogenous secondary metabolites from classified and unclassified *Haliclona* sponges as well as from their symbiotic microorganisms. Most of these substances have only been found in *Haliclona* sponges, and display diverse bioactive properties with potential applications in new drug discovery.

## 1. Introduction

The marine environment is the largest treasure trove of creatures, including plants, animals and microorganisms. People never stop conducting chemical studies of marine organisms owing to their potential capability to produce bioactive secondary metabolites that are potential sources of leads for new drug development. So far over 34,000 articles about marine-derived natural products research have been published [1]. Marine sponges, which are immemorial organisms, are widely distributed around the world and comprise over ten thousand species, most of which them live in the sea, while only one percent are freshwater sponges [2]. These creatures are a great source of natural products with a broad spectrum of biological properties. Numerous sponge-derived chemicals, especial alkaloid compounds, display pharmacological effects, such as didemnin B, cytarabine, trabectedin, vidarabine, etc.

The marine sponge genus *Haliclona* belongs to the family Chalinidae, order Haplosclerida, class Demospongiae [3]. Its skeleton is made up of grids of single spines or a network of spongy fibrous branches without an epidermal skeleton [2]. The *Haliclona* genus consists of over 600 species distributed throughout the world [3]. However, only a few dozen specimens collected from the Pacific Ocean [4], north Indian Ocean [5], Atlantic Ocean [6], and Mediterranean Sea [7] have been chemically investigated. These studies suggest that *Haliclona* sponges are some of the most prolific producers of bioactive secondary metabolites. On the basis of a extensive literature search using SciFinder, this review firstly summarizes all nitrogenous substances from the marine sponge genus *Haliclona* and its symbiotic microbes.

## 2. Bioactive Alkaloids from *Haliclona* Genus

As many as 103 alkaloid secondary metabolites have been isolated and characterized from *Haliclona* sponges since 1994. However, only 32 of them (compounds **1**–**32**) were from seven classified *Haliclona* species including *H. baeri*, *H. cymaeformis*, *H. densaspicula*, *H. exigua*, *H. koremella*, *H. nigra* and *H. tulearensis*, and are respectively introduced in detail according to their biological sources. Other alkaloids (compounds **33**–**103**) from unclassified *Haliclona* sponges are grouped into three types on basis of their chemical structures, namely 3-alkylpyridine, amide and depsipeptide, and miscellaneous alkaloids.

### 2.1. Haliclona Baeri

**Figure 1:**
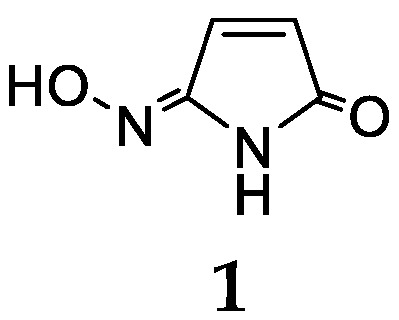
Chemical structure of compound **1** from *Haliclona baeri*.

There is only one report on a chemical study of *Haliclona baeri* collected from the coast of Jongbrii Province (Thailand) [8]. One new nitrogenated compound maleimide-5-oxime (**1**) along with one benzoic derivative and two tetillapyrones was separated from this sponge (Figure 1). The follow-up bioassay tests suggested that compound **1** had weak cytotoxic activity against the human DAOY medulloblastoma cell line at 50 μg/mL [9].

### 2.2. Haliclona Cymaeformis

**Figure 2:**
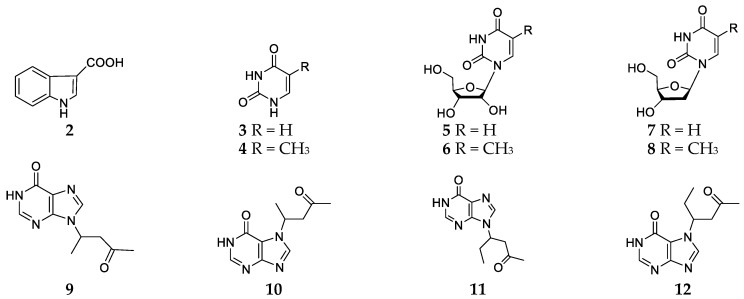
Chemical structures of compounds **2**–**12** from *Haliclona cymaeformis*.

Fractionation of the ethanol extract of *Haliclona cymaeformis* collected from a Xuwen coral reef (Guangdong, China) using silica gel column chromatography led to isolation of eleven alkaloids, including one indole alkaloid **2**, six nucleosides **3**–**8** and four sterols (Figure 2) [10]. Subsequently two pairs of 6-oxypurine regioisomers substituted at the 7 or 9 position (compounds **9**–**12**) were purified from the same specimen (Figure 2) [11].

### 2.3. Haliclona Densaspicula

**Figure 3:**
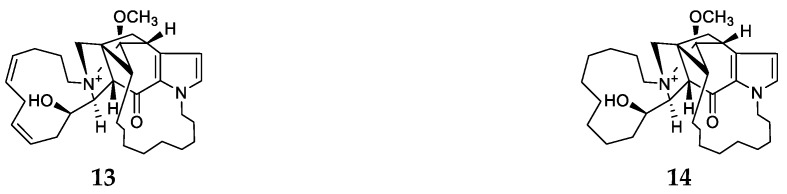
Chemical structures of compounds **13**–**14** from *Haliclona densaspicula*.

Two novel alkaloids with six hexacyclic diamines, densanins A (**13**) and B (**14**) (Figure 3), were found in methanol extract of the sponge *Haliclona densaspicula* from Keomun Island (Korea) and their absolute chemical structures were determined by 1D and 2D NMR spectral analysis and Mosher reactions. Biological evaluation indicated that compounds **13** and **14** possess potent inhibitory effects on lipopolysaccharide-induced nitric oxide production in BV2 microglial cells with IC_50_ values of 1.05 and 2.14 μM, respectively [4,12].

### 2.4. Haliclona Exigua

**Figure 4:**
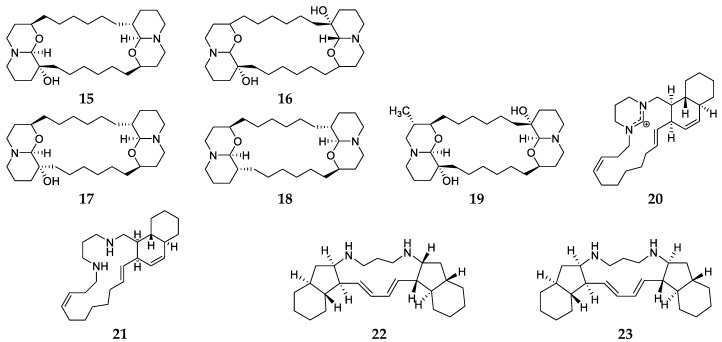
Chemical structures of compounds **15**–**23** from *Haliclona exigua*.

Chemical study of the sponge specimen *Haliclona exigua* collected from the coastal areas of India and Indonesia afforded nine alkaloid derivatives, including xestospongin D (**15**), araguspongins C-E (**16**–**18**), 3α-methylaraguspongine C (**19**), neopetrocyclamines A (**20**) and B (**21**), papuamine (**22**) and haliclonadiamine (**23**) (Figure 4) [5,13,14,15,16,17]. Compound **16** was the most common alkaloid from *H. exigua* and shown to have strong inhibitory activity against human lymphatic filarial parasite B, promastigote and intracellular amastigote forms of *Leishmania donovani*, and anti-fouling and competitive inhibition of NOS [5,15,16,17]. This compound was later purified from an Indonesian sponge *Neopetrosia chaliniformis* and could inhibit zebrafish embryos with a LD_50_ value of 4.3 μg/mL [18]. Compound **22** possessed remarkable cytotoxicity toward human glioblastoma cell line SF-295 with a GI_50_ value of 0.8 μM and **23** could control the transfer of MDA-MB-231 breast cancer cells [14]. Additionally, these alkaloids **22** and **23** obtained from an Okinawan sponge *H. panicea* and were found to inhibit the growth of *Mycobacterium bovis* BCG, *M. intracellulare* and *M. smegmatis* [19,20].

### 2.5. Haliclona Nigra

**Figure 5:**
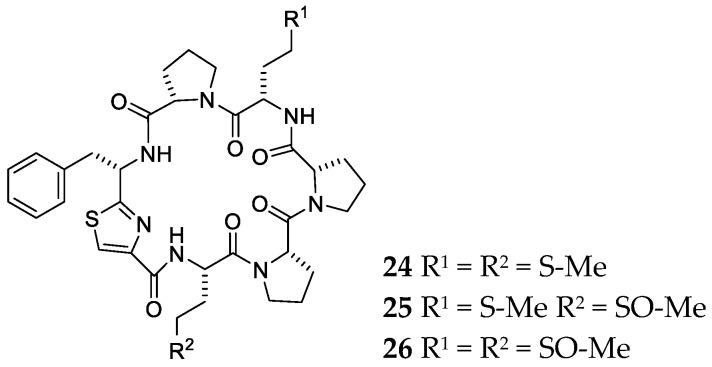
Chemical structures of compounds **24**–**26** from *Haliclona nigra*.

Fractionation of the aqueous extract of the marine sponge *Haliclona nigra* collected from northern coast of Papua New Guinea resulted in the discovery of two new hexapeptides, haligramides A (**24**) and B (**25**), together with waiakeamide (**26**) (Figure 5) [21]. Their chemical structures and configurations were elucidated by extensive NMR analyses and oxidative reactions.

### 2.6. Haliclona Tulearensis

**Figure 6:**
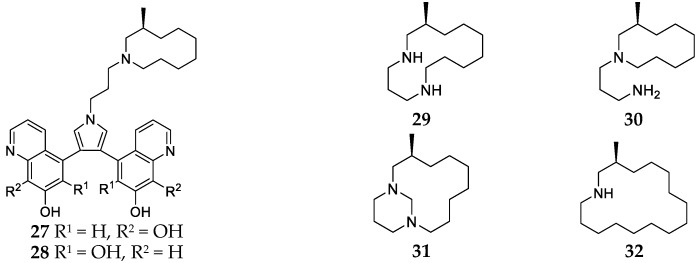
Chemical structures of compounds **27**–**32** from *Haliclona tulearensis*.

Five new alkaloids, halitulin (**27**), halic isolorensin (**29**), isohaliclorensin (**30**), haliclorensin B (**31**) and haliclorensin C (**32**) together with isohalitulin (**28**) (Figure 6), were purified from the sponge *Haliclona tulearensis* collected from Sodwana Bay (South Africa) [22,23,24]. Compound **29** was a novel diamino derivative possessing an azacyclodecane ring, and exhibited strong cytotoxicity against P-388 mouse leukemia cells with an IC_50_ value of 0.1 mg/mL [21]. In vitro biological evaluation results suggested that compounds **27**, **29**, **31** and **32** had significant cytotoxicity against P-388 with IC_50_ values of no more than 0.1 μg/mL [23,24].

### 2.7. Other Haliclona Spps.

Until now, up to 71 nitrogenated compounds **33**–**103** have been discovered from unidentified marine sponge species of *Haliclona*. According to their chemical structures, these substances can be grouped into three classes, including 3-alkylpyridines, amides and depsipeptides, and miscellaneous alkaloids.

#### 2.7.1. 3-Alkylpyridines

3-Alkylpyridine analogs with linear and cyclic frameworks such as navenones, halitoxins, niphatynes, niphatesines, haminols, viscosamine, etc [25] are the most common alkaloids isolated from the marine sponge genus *Haliclona* sponges. These substances possess pronounced biological activities.

**Figure 7:**
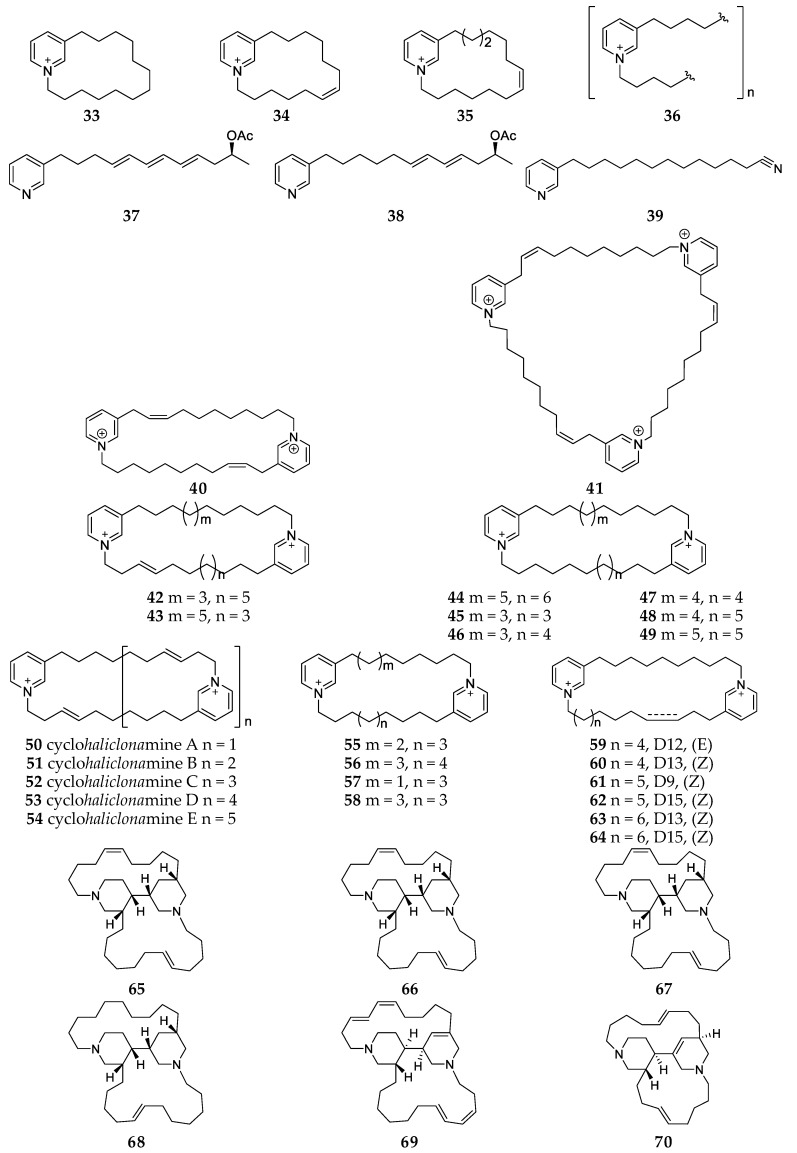
Chemical structures of compounds **33**–**70** from unclassified *Haliclona* sps.

Chemical study of a marine sponge *Haliclona* sp. from New Zealand led to isolation of haliclocyclin C (**33**) and two new alkaloids dehydrohaliclocyclins C (**34**) and F (**35**) (Figure 7), which were the first examples of cyclic 3-alkylpyridinium alkaloid (3-APA) monomerz with an unsaturated alkyl chain [26]. An anti-fouling mixture of poly 3-alkylpyridinium salts (**36**) as well as haminols (**37-38**) was firstly isolated from the methanol extract of *Haliclona* sp. collected in Terra Nova Bay, Ross Sea (Antarctica) (Figure 7) [27]. From an Indonesian sponge *Haliclona* sp. no. 95546, a new alkaloid, 3-dodecyl- pyridine (**39**) bearing a terminal cyano group, was purified and found to possess moderate in vitro cytotoxicity against tumor cell lines A549, MCF7 and Hela with the IC_50_ values of 41.8, 48.4, 33.2 μM, respectively. Two new alkaloids **40**–**41** with dimeric and trimeric 3-APA moieties were isolated from the methanolic extract of the sponge *Haliclona* sp. collected in the Pacific coast of Guatemala (Figure 7) [28]. Bioassay results indicated that compounds **40** and **41** had low cytotoxic effect on murine macrophage J774.A1 and fibrosarcoma WEHI-164 cell lines and human epithelial kidney HEK-293 with IC_50_ > 20 μg/mL.

Haliclocyclamines A–C (**42–44**), three new cyclic bis-1,3-APA derivatives, found together with five analogs cyclostellettamines A–C (**45**–**47**), E (**48**) and F (**49**), were separated from the EtOH extract of *Haliclona* sp. collected at Manado in Indonesia (Figure 7) [29,30]. Compounds **44** and **49** could inhibit vaccinia H-1-related phosphatase (VHR) at 17–18 μM and compound **45** possessed cytotoxic effect on HeLa cells with an IC_50_ value of 0.9 μg/mL and P388 cells with an IC_50_ value of 1.06 μg/mL. A heterogeneous and inseparable mixture of five cyclic 3-APAs, cyclohaliclonamines A-E (**50-54**), was isolated from an Okinawa sponge *Haliclona* sp., which compounds **52**–**54** were the first tetrameric, pentameric and hexameric 3-APAs from natural sources (Figure 7) [25]. By solvent partition, Sephadex LH-20 gel permeation and HPLC, ten cyclic bis-1,3-APA derivatives (**55**–**64**) were separated from the MeOH extract of *Haliclona* sp. collected at the shore of Jeju Island (Korea) and characterized using combined NMR and FAB-MS/MS analyses (Figure 7) [31]. Compounds **56** and **62**–**64** had moderate antibacterial activities against *Staphylococcus aureus* with the same MIC values of 12.5 μg/mL, and compound **63** exhibited the highest cytotoxic against A549 cell-line with a LC_50_ value of 14.7 μg/mL. Six tetracyclic alkaloids, haliclonacyclamines A-D (**65**–**68**) and halicyclamines A (**69**) and B (**70**) consisting of two 3-alkyl hexahydropyridine units were separated from *Haliclona* sp. grown on acroporid coral substrate on the Heron Island (Australia), and compounds **65** and **66** could inhibit the growth of leukemia P-388 cells with IC_50_ values of 0.8 and 0.6 μg/mL, respectively (Figure 7) [32,33]. Halicyclamines A (**69**) derived from the Indonesian sponge *Haliclona* sp. 05A08 was shown to possess inhibitory effect on inosine-phosphate dehydrogenase at 1 μg/mL and anti-microbial activity against *Mycobacterium smegmatis* and *M. bovis* BCG with MIC values of 2.5 and 1.0 μg/mL, respectively [34]. Compound **70** showed cytotoxicity against HeLa cells with an IC_50_ value of 12 μM and inhibitory effect on chymotrypsin and caspase with IC_50_ values of 0.42 and 0.48 μM, respectively [35].

#### 2.7.2. Amides and Depsipeptides

**Figure 8:**
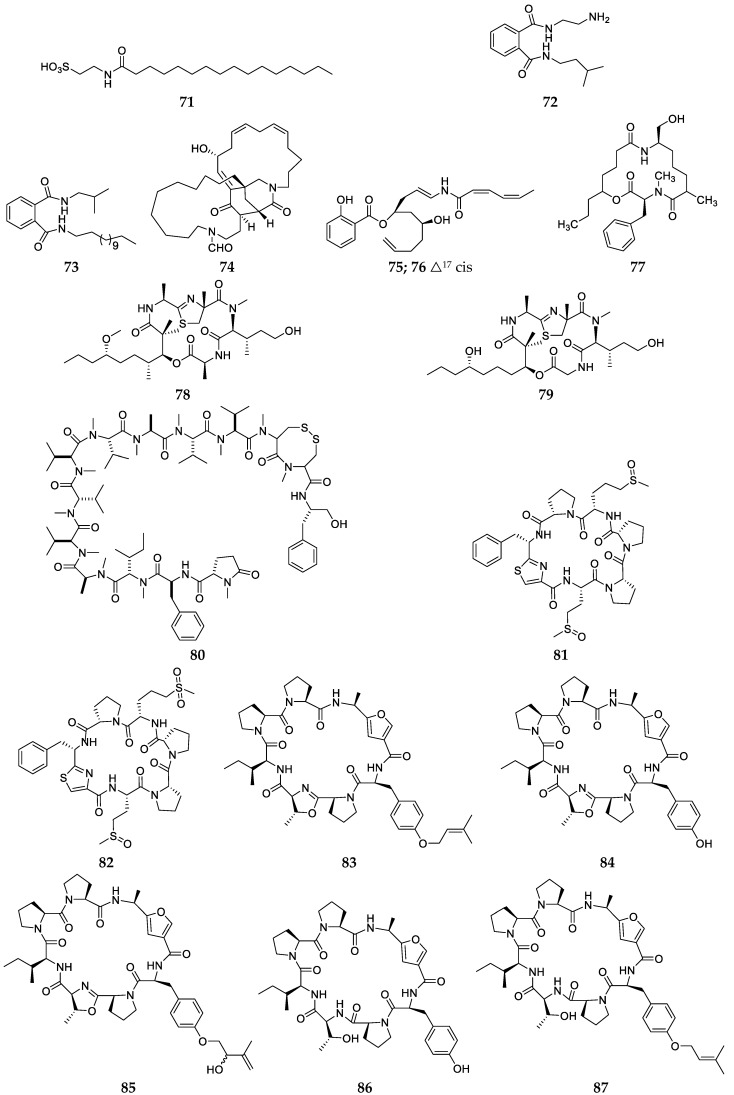
Chemical structures of compounds **71**–**87** from unclassified *Haliclona* sps.

Three novel amides, 2-palmitamidoethane sulfonic acid (**71**), *N*^1^-(2-aminoethyl)-*N*^2^-isopentylphthalamide (**72**) and *N*^1^-isobutyl-*N*^2^-tridecylphthalamide (**73**), were obtained from a *Haliclona* sp. sponge collected off the coast of Hainan Island (China) (Figure 8) [36,37]. Haliclonin A (**74**), a new macrocyclic diamide from the Korean-derived sponge *Haliclona* sp. exhibited moderate antibacterial activity with a MIC value of 6.25 µg/mL against *Bacillus subtilis*, and cytotoxicity against the K562 leukemia cell line with an IC_50_ of 15.9 µg/mL (Figure 8) [38]. Chemical analysis of a Rottnest Island-derived sponge *Haliclona* sp. afforded two new olefinic amides, salicylihalamides A (**75**) and B (**76**) (Figure 8) [39]. Chemical investigation of a *Haliclona* sp. sponge specimen from the Vanuatu Islands afforded three new amides, haliclamide (**77**), halipeptins A (**78**) and B (**79**) (Figure 8) [40,41]. The structures of compounds **78** and **79** were later revised and corrected by total chemical synthesis [42]. Compound **77** exhibited in vitro anti-tumor activity against human bronchopulmonary non-small-cell-lung-carcinoma line NSCLC-N6 with an IC_50_ value of 4.0 µg/mL while **78** displays potent anti-inflammatory effect. One new depsipeptide, kendarimide A (**80**), was isolated and characterized from a Sulawesi Island-derived *Haliclona* sp. Sponge (Figure 8) [43]. A MTT assay suggested that this chemical had 87% growth inhibition on multi-drug resistance (MDR) cell line KB-C2 cells in the presence of 0.1 mg/mL colchicine. Two cyclic hexapeptides, waiakeamide (**81**) and its sulfone derivative **82**, and five cyclic heptapeptides, the haliclonamides A-E (**83**–**87**) (Figure 8), were sequentially purified from a Palau-derived *Haliclona* sp. sponge [44,45,46]. Bioassay results showed that these peptides had potent antifouling activity at the concentration of 100 ppm, except for compounds **84** and **86**.

#### 2.7.3. Miscellaneous Alkaloids

**Figure 9:**
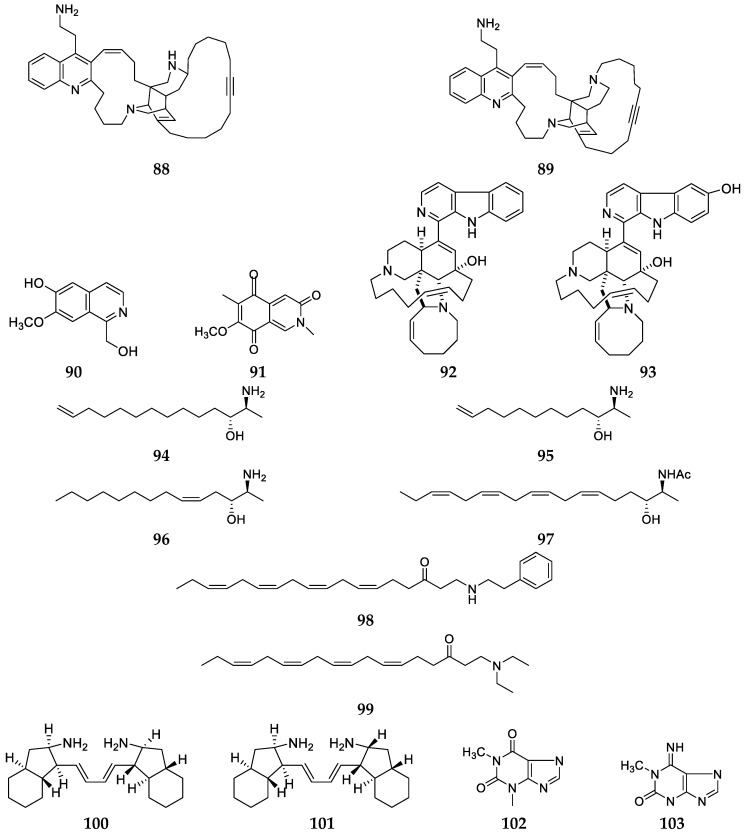
Chemical structures of compounds **88**–**103** from unclassified *Haliclona* sps.

A new cytotoxic polycyclic alkaloid njaoamine I (**88**) containing a quinoline system and a known cytotoxic compound njaoamine G (**89**) were detected in the methanol extract of a *Haliclona* (*Reniera*) sp. sponge collected from Okuza Island (Tanzania) (Figure 9) [47]. Two isoquinoline alkaloids, 1-hydroxymethyl-7-methoxyisoquinolin-6-ol (**90**) and mimosamycin (**91**) (Figure 9), were purified from a *Haliclona* sp. sponge collected at Jessie Beazley Reef (Philippines) [48]. Interestingly, compound **91** was also produced by the marine sponge *Cribrochalina* and had strong cytotoxic effect on human tumor cell lines LOX, OVCAR-3 and HeLa cells with IC_50_ values of 10, 10, 2.6 μg/mL, respectively [48,49]. Manzamines A (**92**) and Y (**93**) (Figure 9), two unusual alkaloids with β-carboline and isoquinoline skeletons and a 13-element dense, *N*-containing polycyclic structure unit, were isolated and characterized from two specimens of the sponge *Haliclona* sp., which were respectively collected from Manzamo and Iriomote Island [50,51]. Compound **92** could strongly inhibit the growth of mouse P-388 cells with an IC_50_ value of 0.07 μg/mL while **93** showed weak cytotoxicity on KB cells (IC_50_ = 7.3 μg/mL).

Four antifungal amino alcohols, halaminols A–D (94–97) (Figure 9), were purified from a *Haliclona* sp. sponge grown on the Great Barrier Reef and their relative configurations were deduced from the NMR characteristics of oxazolidinone derivatives and absolute configurations were determined by their MPA esters [52]. Two new uncommon amino ketones, (6Z,9Z,12Z,15Z)-1-[(2-phenyl-ethyl)amino]octadeca-6,9,12,15-tetraen-3-one (98) and (6Z,9Z,12Z,15Z)-1-(diethylamino)octadeca-6,9,12,15-tetraen-3-one (99) (Figure 9), were separated from an unclassified *Haliclona* sponge collected from Weizhou Island (Guangci, China) [53]. Chemical study of the EtOH extract of a Haliclona sp. sample collected from Iriomote Island (Japan) afforded two new haliclonadiamine derivatives, halichondriamine C (100) and 1-epi-halichondriamine C (101) along with papuamine (22) and haliclonadiamine (23) (Figure 9) [19]. Compounds 100 and 101 could inhibit the growth of *Mycobacterium bovis* BCG with the same MIC values of 0.5 μg/mL and *M. intracellulare* with MIC values of 1.0 and 0.5 μg/mL, respectively. Additionally, two purine derivatives, 1,3-dimethylpurine (102) and 1,3-dimethyl-6-imino (103) (Figure 9), were separated from a *Haliclona* sp. sponge grown on Hainan Island (China) [54].

## 3. Bioactive Alkaloids from *Haliclona*-Derived Microbes

**Figure 10:**
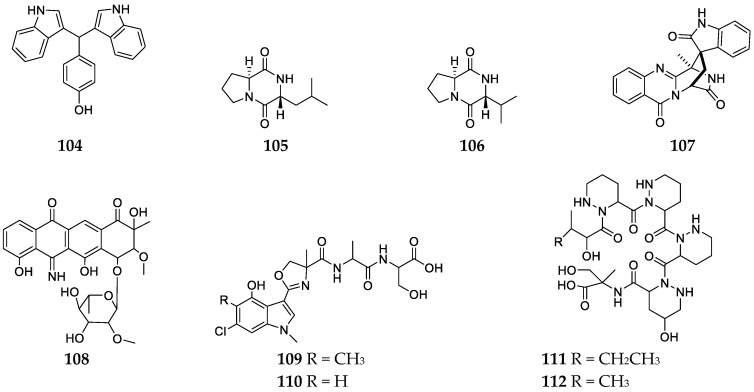
Chemical structures of compounds **104**–**112** from *Haliclona*-derived microbes.

Marine sponges are important hosts for a large community of microorganisms, which are shown to be great producers of secondary metabolites [55]. However, only eight alkaloids **104**–**112** have been separated from *Haliclona* sponge-derived microbes until now (Figure 10). Chemical analysis of the ethyl acetate extract of the strain *Bacillus megaterium* LC3CS2 symbiont of the sponge *Haliclona oculata* collected from Son Cha Peninsula (Vietnam) afforded three anti-microbial agents: 7,7-bis(3-indolyl)-*p*-cresol (**104**), cyclo-(Pro-Leu) (**105**) and cyclo-(Pro-Val) (**106**) (Figure 10) [56]. These chemicals had antimicrobial activities against *Vibrio vulnificus*, *V. parahaemolyticus* and *Trichophyton mentagrophytes* with MIC values ranging from 0.05 to 5.0 µg/mL. Compound **104,** formerly obtained from a marine sponge *Hyatell*-derived microbe *Vibrio* sp. was shown to inhibit the growth of *Bacillus cereus* and *Micrococcus luteus* with MIC values of 0.5 and 0.005 µg/mL, respectively [56,57]. Alantrypinone (**107**) along with lovastatin, methyl ester of lactone ring-opened monacolin K, terrein, territrems B and ergosterol was separated from a F62 fungal strain associated with the sponge *Haliclona simulans* collected from the South China Sea (Figure 10) [58]. Screening of symbiotic strains from the marine sponge *Haliclona* sp. collected from the sea shore of Tateyama city (Japan) led to the discovery of four new *Streptomyces* strains [59]. Later chemical investigation of strains GE-23 GE-26 and SC-24 afforded five new alkaloids JBIR-30, -34, -35, -39 and -40 (**108**–**112**) (Figure 10). However, none of these compounds had potent cytotoxic effects on human cervical carcinoma HeLa cells and malignant pleural mesothelioma ACC-MESO-1 cells.

## 4. Conclusions

In summary, 112 nitrogenous secondary metabolites have been isolated and characterized from the marine sponge genus *Haliclona* and its derived microbes till now. Only five alkaloids (compounds **16**, **22**, **23**, **91** and **104**) were separated from other organisms. Therefore, this indicates that *Haliclona* sponges are some of the most prolific sources of exclusive bioactive alkaloids despite the fact only a handful of classified species had been chemically investigated. It is well-known that marine organisms have served as a primary source of bioactive natural products during the past several decades. Nowadays, however, a rapid decrease in the speed of discovery of new compounds from Nature strongly necessitates new research strategies and approaches. Microorganisms are ubiquitous in the ocean owing to their stronger adaptability. During long co-evolution with marine sponges, symbiotic microbes maybe play important physiological and ecological roles in promoting host growth and increasing the resistance to predators and omnivores by excreting toxic metabolites. Therefore, more efforts should be made to explore and identify unknown *Haliclona* sponges and their derived symbiotic microbes and to carry out chemical studies for the discovery of novel therapeutical agents.
